# A modified direct anterior approach for primary total hip arthroplasty: surgical technique

**DOI:** 10.1186/s13018-025-06397-5

**Published:** 2025-10-22

**Authors:** Raffaele Iorio, Federico Corsetti, Simone Fenucci, Edoardo Viglietta, Yuri Gugliotta, Filippo Migliorini, Nicola Maffulli

**Affiliations:** 1 Faculty of Medicine and Psychology, University La Sapienza, 00185 Roma, Italy; 2 Department of Trauma and Reconstructive Surgery, University Hospital of Halle, Martin-Luther University Halle-Wittenberg, 06097 Halle (Saale), Germany; 3 Department of Orthopaedic and Trauma Surgery, Academic Hospital of Bolzano (SABES-ASDAA), 39100 Bolzano, Italy; 4 Department of Life Sciences, Health, and Health Professions, Link Campus University, 00165 Rome, Italy; 5 School of Pharmacy and Bioengineering, Keele University Faculty of Medicine, Stoke on Trent, ST4 7QB UK; 6 Centre for Sports and Exercise Medicine, Barts and the London School of Medicine and Dentistry, Mile End Hospital, Queen Mary University of London, London, E1 4DG UK

**Keywords:** Surgical exposure, Traction table, Lateral femoral cutaneous nerve, Capsulotomy, Technical modification, Surgical reproducibility, Complication avoidance

## Abstract

The direct anterior approach (DAA) for total hip arthroplasty is increasingly adopted because of its muscle-sparing nature and potential for faster recovery. However, its use is often limited by the need for a traction table, dedicated instrumentation, and a demanding learning curve. We present a simplified modification of the DAA that can be performed on a standard operating table without specialised equipment. The technique preserves the Hueter interval but introduces specific refinements, including a slightly lateralised skin incision to reduce the risk of lateral femoral cutaneous nerve injury and a U-shaped capsulotomy to facilitate acetabular exposure and en bloc soft tissue management. These adjustments eliminate traction-related complications, reduce reliance on additional staff and costly instruments, and streamline the operative workflow. In our experience, this modified approach has been reproducible across a wide range of patient phenotypes, including obese or muscular individuals, with a low incidence of typical complications. This variant therefore preserves the benefits of the anterior approach while addressing many of its practical limitations, offering a versatile, safe, and accessible option for primary and selected revision total hip arthroplasty.

**Supplementary Information:**

The online version contains supplementary material available at 10.1186/s13018-025-06397-5.

## Introduction

Total hip arthroplasty (THA) is a highly successful procedure [1]. Several surgical approaches have been described, each becoming more or less fashionable over time, with none exhibiting clinically relevant differences in mid- to long-term outcomes [2, 3]. The goal of each approach is to provide optimal visualisation of the femur and acetabulum, minimise complications, and improve patient outcomes [4–7]. The Direct Anterior Approach (DAA), first described by Carl Hueter in 1881 [7] and subsequently refined by Smith-Petersen et al. [8], has gained popularity in recent years [9, 10]. By developing the Hueter interval between the tensor fascia latae (TFL) and sartorius muscles, the DAA avoids muscle detachment from bone, thereby reducing muscle damage, allowing earlier restoration of gait kinematics, faster recovery, and lower dislocation rates [11–14]. Patients undergoing THA with the DAA may also experience less postoperative pain and reduced need for opioids [15–17]. Additionally, the approach requires a smaller incision, which may improve cosmetic outcomes [18]. Although the original description of the DAA did not include the use of a traction table, most widely adopted variants have incorporated it to improve femoral exposure and mobilisation [11, 19]. However, reliance on the traction table is associated with specific complications, including perioperative fractures [20], and necessitates the presence of dedicated staff to manage table adjustments intraoperatively. Moreover, it requires specialised instruments designed exclusively for the anterior approach, increasing procedural costs. A longer learning curve also characterises the DAA compared to more traditional methods [21], which limits its feasibility in smaller-volume centres where case numbers are restricted. Despite these challenges, several studies have consistently demonstrated the advantages of the DAA in reducing soft-tissue trauma, particularly to the gluteal and abductor muscles. MRI-based evaluations confirm a lower degree of muscle damage compared with other approaches, which translates into reduced blood loss, shorter hospital stay, and improved patient comfort [22, 23]. Nevertheless, highly muscled or obese patients remain less suitable candidates for this approach [24]. In light of these considerations, we report a simplified modification of the DAA that does not require a traction table or specialised equipment, aiming to maintain the benefits of the anterior approach while overcoming its practical limitations. This study was structured and reported in accordance with the SUPER (Surgical techniqUe rePorting checklist and standaRds) guidelines from the EQUATOR Network, ensuring methodological transparency and reproducibility [26]. 

## Surgical technique

This variant of the direct anterior approach (DAA) was developed to simplify the procedure and reduce the risk of complications specific to the approach. It does not require a traction table or dedicated staff for its management, and no exclusive instrumentation is necessary. Except for offset reamers, which may improve convenience but can be replaced with standard straight reamers, all instruments are commonly available in standard orthopaedic theatres.

Although the literature reports reduced indication for the DAA in obese or highly muscled patients, in our experience, this modification can be safely and reproducibly applied across all patient phenotypes, including those with complex deformities. With appropriate extensions, it can also be employed in revision cases and other demanding procedures, thereby representing a versatile, safe, and broadly applicable technique. In our experience, none of the specific complications commonly described for the DAA have been observed with this variant. While lateral femoral cutaneous nerve neuropraxia is frequently reported in the literature as a typical complication, in our hands, it has been a rare finding and has always resolved spontaneously with conservative management [20, 27]. 

### 1. Patient positioning

The patient is positioned supine on the operating table; the pelvis is thus stable, and the hip to be operated on can be adducted and extended for adequate femoral exposure. Skin preparation is undertaken using double disinfection with chlorinated and iodised fluid, and transparent plastic drapes are used to prepare and protect the surgical field.

### 2. Incision

**Fig. 1:**
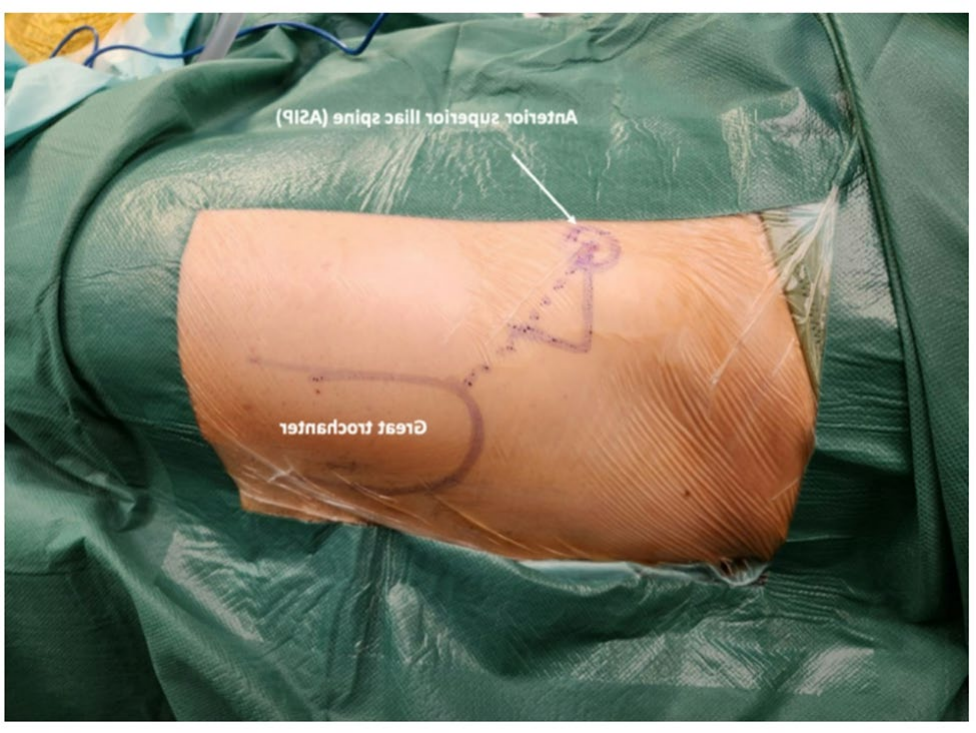
The direct anterior access: The incision point is at middle third of the junction with the great trochanter and the anterior superior iliac spine.

**Fig. 2:**
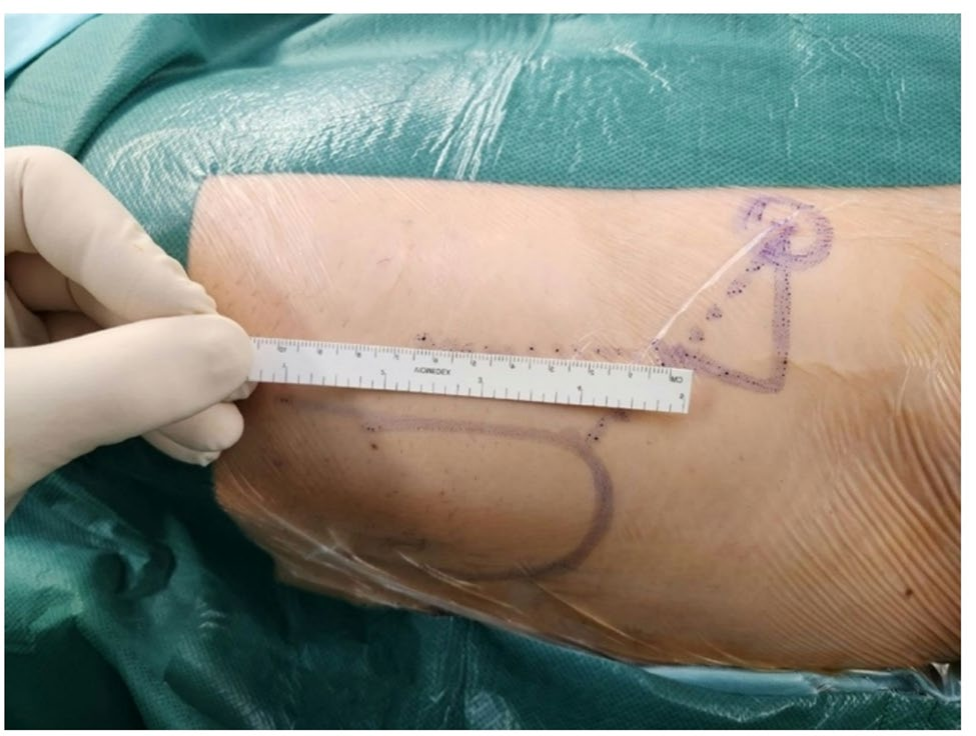
Skin incision: A 8–10 cm skin incision directing towards the fibula head is required to get started

**2a.** The skin incision starts at the middle third of the greater trochanter, 3 cm lateral and 3 cm distal to the anterosuperior iliac spine, directing it towards the head of the fibula. (Figure 1) the initial incision is 8–10 cm, and can be extended if necessary.(Figure 2).

**Fig. 3:**
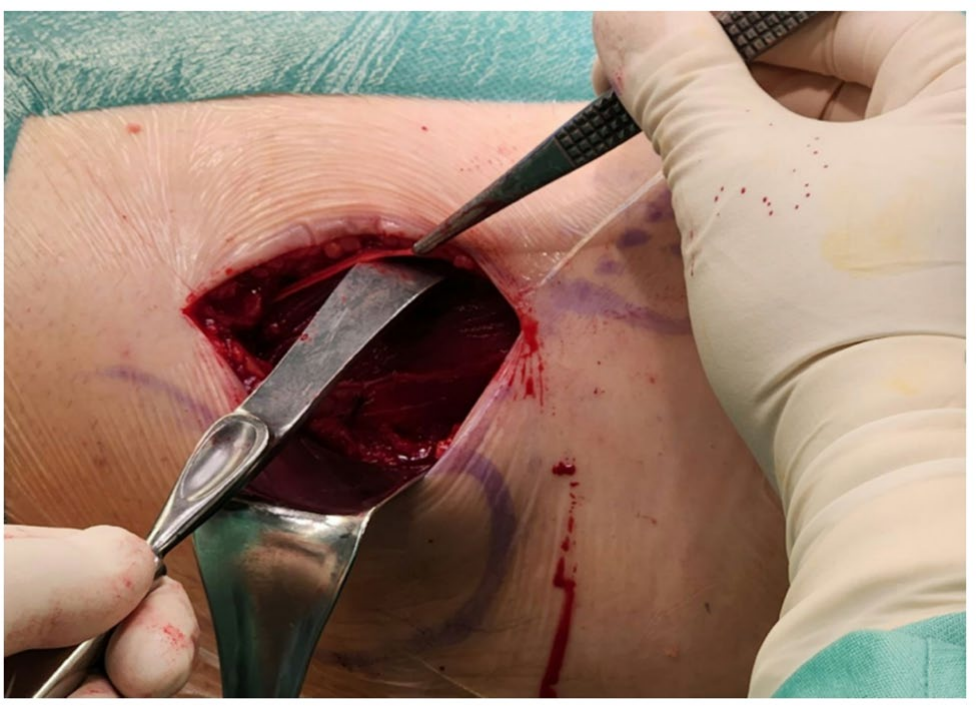
The Hueter space: The tensor fascia latae fascia is gently exposed, peeling back. The TFL fascia is dissected to its edge, developing the Hueter space.

**2b.** Following the direction of the muscle fibres, the fascia over the tensor fasciae latae (TFL) is incised lateral to its edge, developing the space between the TFL and sartorius. Blunt dissection of the fascia over the femoral neck is performed, retracting the adipose tissue and muscle fibres. (Figure 3) a Hohmann bevel retractor is placed superiorly behind the femoral neck. Another Hohmann retractor is placed posterior to the greater trochanter to retract the TFL laterally. Finally, a Richardson-Eastman retractor is used to retract the rectus femoris muscle laterally.

**Fig. 4:**
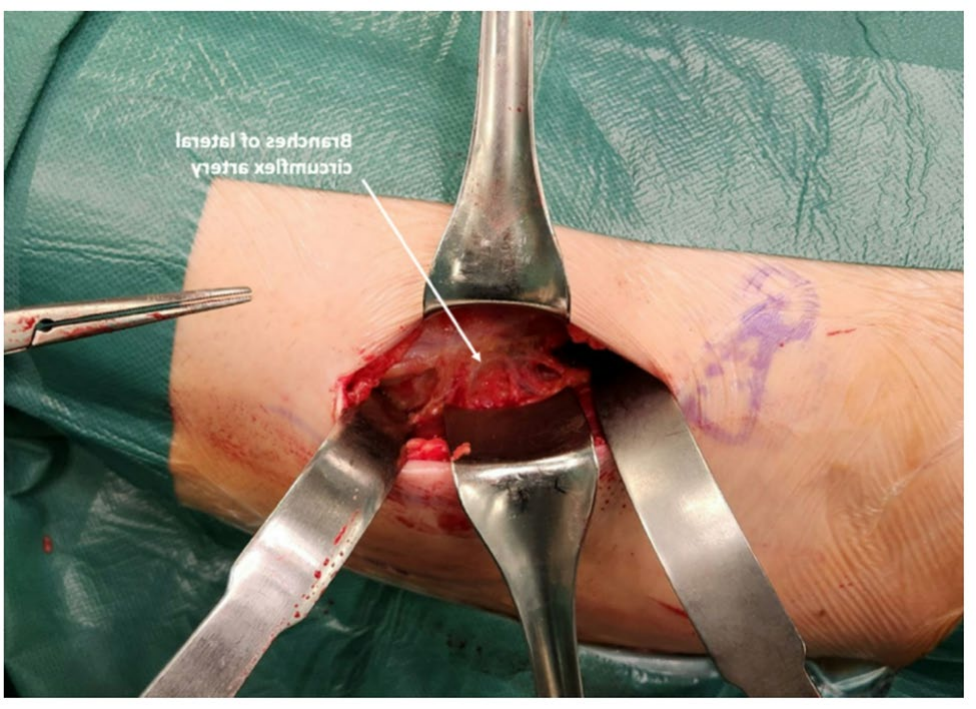
Exposing anterior capsule: The ascending branches of the lateral circumflex artery lie between the TFL and sartorius, and are carefully coagulated. Then, the anterior capsule is exposed

**2c.** The ascending branches of the lateral circumflex artery lie between the TFL and Sartorius, and are carefully coagulated (our preference) or ligated. (Figure 4) once the fat and soft tissue are cleared, the anterior aspect of the hip joint capsule is exposed, and the interval between the capsule and gluteus minimus at the superior femoral edge is visualised.

**2d.** The soft tissue plane medially over the capsule of the inferior aspect of the femoral neck is developed by gently elevating the rectus femoris and the Iliocapsularis muscles. A blunt Hohmann retractor is placed extracapsularly inferiorly on the femoral neck.

**Fig. 5:**
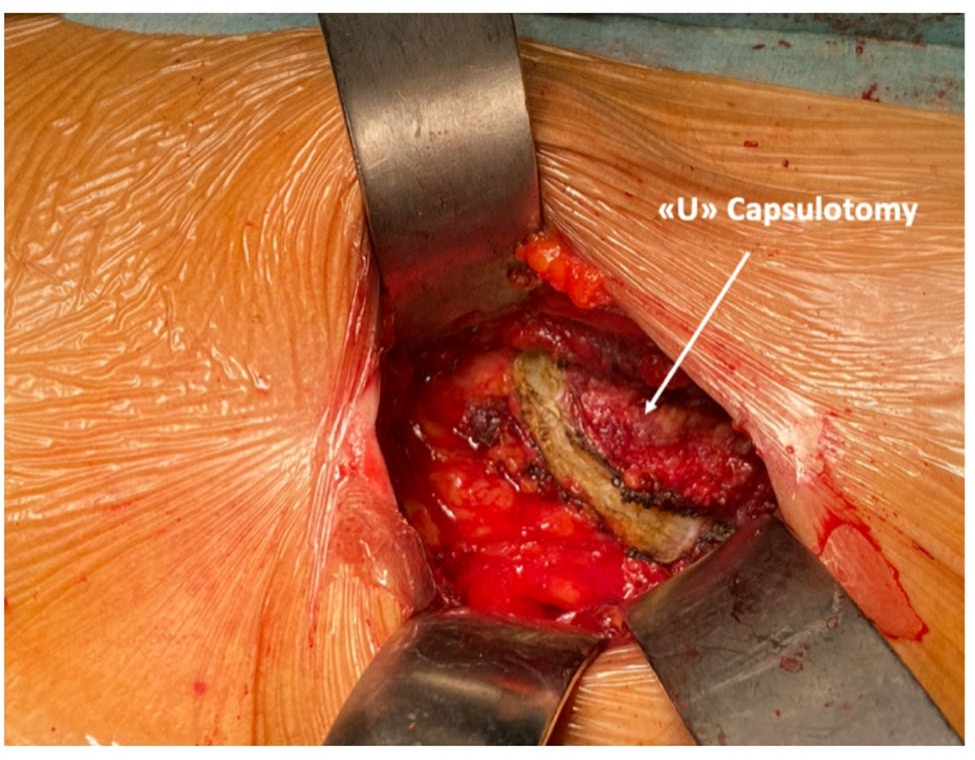
The “U” capsulotomy: A“U” capsulotomy is performed to expose the femoral head. The curved incision preserves more capsular tissue

**2e.** The femoral neck is exposed using a “U “capsulotomy. (Figure 5) a curved incision is performed to preserve capsular tissue, which is then dissected from the femoral neck surface. A strong Vycril 2 stay suture is applied to the capsular flap to help lift the rectus muscle and overlying soft tissues. Then, the superolateral and inferomedial retractors are repositioned inside the capsule.

### 3. Femoral head osteotomy

**3a.** Using the greater and lesser trochanters as landmarks, an osteotomy of the femoral neck is performed, keeping the two Hohmann retractors in situ. A Hohmann retractor protects the vastus lateralis. The femoral neck osteotomy is performed 1 cm cranial to the lesser trochanter. The cutting direction targets the apex of the greater trochanter, maintaining a 45-degree angle. A double-cut osteotomy of the neck about 8–10 mm thick with parallel cuts produces a slice of bone, which is duly removed. After the femoral neck osteotomy, a corkscrew femoral head extractor is used to remove the head of the femur, taking care to protect the TFL muscle.

### 4. Acetabular exposure

**4a.** The limb operated on is first moved to the “4 position up” to externally rotate the femur and measure the distance from the lesser trochanter to the calcar cut. Flexing the knee, the leg is positioned above the contralateral one.

**4b.** A Hohmann retractor is placed at the 3 o’clock position over the posterior rim of the acetabulum.

**4c.** A second Hohmann retractor is placed at the 9 o’clock position over the acetabular roof to retract the overlying soft tissue.

**4d.** A third Hohmann retractor is placed at the 6 o’clock position below the transverse ligament to expose the acetabulum to retract the inferior hip capsule and the iliopsoas tendon at the 6 o’clock position. A fourth retractor may be placed at the 12 o’clock position, supero-anteriorly to enhance acetabular exposure.

### 5. Acetabular reaming and cup implantation

**Fig. 6:**
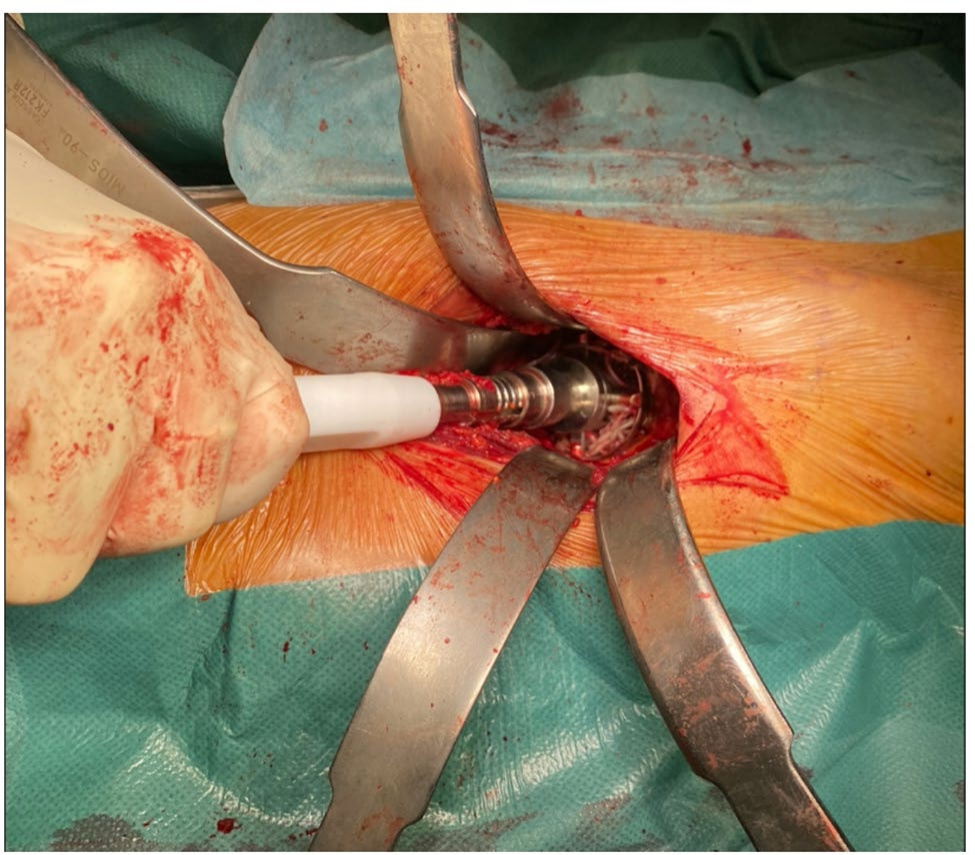
Acetabular exposure: At least three Hohmann retractors are required for proper acetabular exposure. Identification of acetabular edge and the use of offset handlers facilitate acetabular reaming

We suggest using offset acetabular reamers and offset cup insertion handles to facilitate acetabular reaming and cup positioning. (Figure 6) to correctly position the acetabular components, visualisation of the acetabular edge is necessary. The residual capsular tissue and soft tissue in the fovea are then carefully removed. Using offset reamers, the acetabular cavity is prepared to the desired size, and an offset handle allows proper positioning of the acetabular cup. The cup is positioned with the anteversion and inclination angles calculated during pre-operative planning.

### 6. Femoral Preparation

**6a.** The residual capsule over the trochanteric fossa is removed using an electrocautery to visualise the femoral canal better. In case of insufficient exposure, further medial release can be performed, taking care not to detach the piriformis tendon from its fossa.

**6b.** The leg is manoeuvred in the figure of “4 position down”, placing it below the contralateral, with an assistant keeping the leg adducted and externally rotated. The knee is extended during the preparation of the femoral canal.

**Fig. 7:**
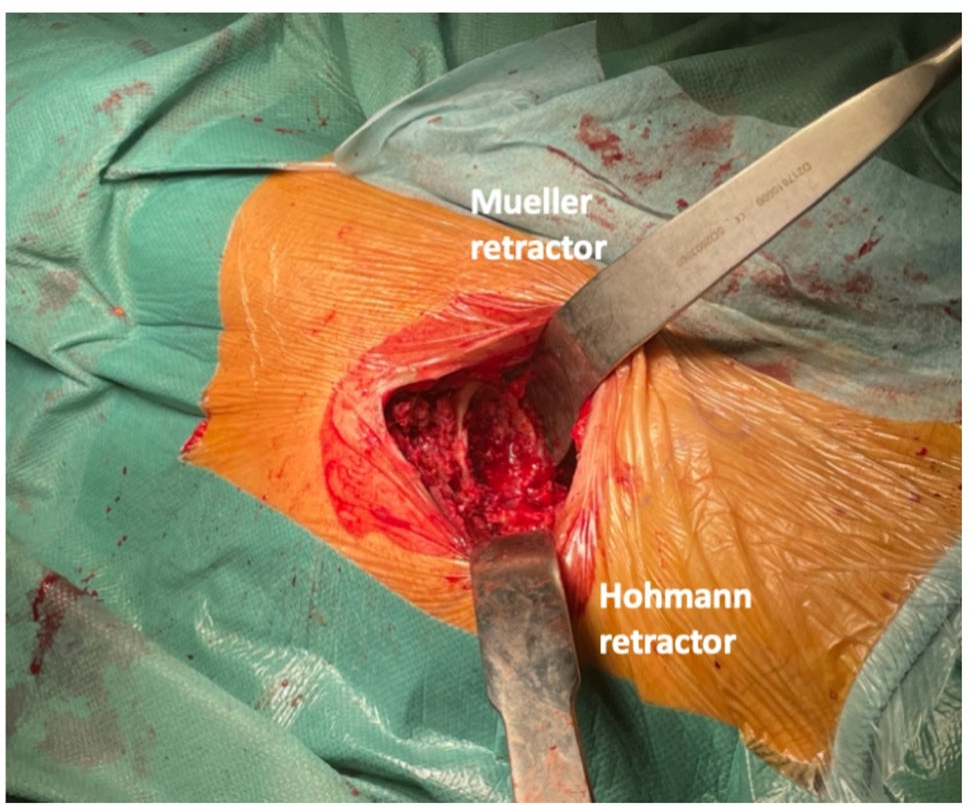
The femoral broaching: Easy access for femoral broaching is obtained positioning a mueller retractor medially and a curved Hohmann retractor laterally to femoral neck. Broaches are placed parallel to the lateral femoral cortex for reaming of the femoral canal. The lesser trochanter is an helpful reference for the correct rotation

**6c.** A Muller femoral retractor is placed medial to the femoral neck to allow full access to the femur for broaching and stem implantation. Another curved “horned” retractor is positioned laterally to the femoral neck, retracting the hip abductor muscles to separate the hip capsule and muscle. (Figure 7) when performing the capsular release, attention should be paid to release the posterolateral area to allow anterior translation of the femur. A bone hook in the femoral canal can help during the release.

### 7. Femoral canal reaming and stem implantation

The femoral canal is located using a femoral canal finder rasp and broached using progressively larger broaches, on offset handles. The reference point during broaching is the lateral cortex of the femur. The broaches are placed parallel to the lateral cortex of the femur to track the direction of the medullary canal. The lesser trochanter should be used as a reference for correct rotation. Broaching is continued to the pre-planned femoral stem size and stops when the broach used is stable in the canal, in the absence of rotational or translational movements.

### 8. Implant reduction

The reduction manoeuvre is performed with the lower limb extended. An assistant pulls and intrarotates the limb while the surgeon gently guides the femoral head into the acetabular cup with a pusher.

### 9. Stability tests

**Fig. 8:**
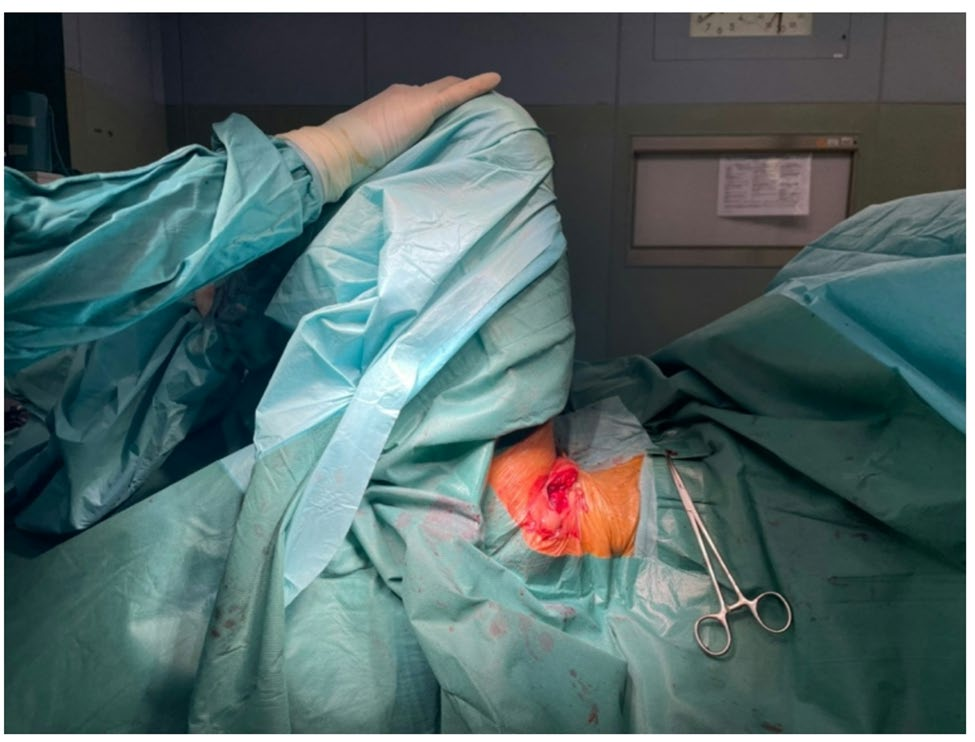
The range of motion evaluation and stability test: Range of motion is tested in maximum hip flexion,intra- and extra-rotation. Anterior stability is tested extending the patient’s hip with the knee flexed

Once the implant is located in its final position, with the patient supine and the leg extended, the range of motion is tested in maximum hip flexion, as well as intra- and extra-rotation. The anterior stability of the implant is tested with the hip in extension and the knee flexed. (Figure 8) these movements should produce no dislocation. Leg length is assessed by comparing the position of the medial malleolus to the contralateral one.

**Tips**.

- U Capsulotomy: A curved capsulotomy allows the preservation of the capsular tissue, functional to retract the overlying soft and muscular tissue en bloc. In this way, the acetabulum is fully exposed for ease of reaming.
- Sparing the lateral femoral cutaneous nerve (LFCN): The skin incision is slightly lateral to the one classically recommended for DAA [9, 23], over the TFL. The location of the incision reduces the risk of iatrogenic injury of the LFCN.
- Femoral medial release: Additional medial release protects the tensor fascia lata during reaming of the femoral canal.

## Postoperative management

In the absence of complications, patients undergoing THA with the anterior approach have no contraindications to immediate full weight-bearing. Verticalization and assisted ambulation are initiated on the first postoperative day. No restrictions are imposed on the range of motion, and patients are not advised to use preventive or assistive devices such as shoehorns or toilet seat raisers. The rehabilitation program does not differ from the standard pathway for THA patients. At our institution, the average length of stay is three nights, after which rehabilitation is predominantly home-based, supported by daily physiotherapy sessions. Follow-up is scheduled at 15 days, 1 month, 3 months and 6 months postoperatively. No braces or patient-specific rehabilitation devices are required.

## Discussion

The direct anterior approach (DAA) has gained increasing popularity over the last decade. It is considered muscle-sparing, associated with reduced postoperative pain, and allows faster recovery of unaided walking in the short term [16, 17]. Nevertheless, evidence supporting its superiority remains limited, as most available data derive from observational or retrospective single-centre studies with heterogeneous reporting of surgical technique, perioperative management, and follow-up [12, 25, 26]. Several randomised and prospective studies have shown that the DAA offers earlier recovery of gait mechanics, reduced perioperative blood loss, and improved short-term function compared with other approaches. However, these benefits tend to equalise in the medium to long term [27–32]. However, the approach is associated with a steep learning curve, often requiring years of experience to achieve proficiency [33, 34], and with inherently longer operative times, although these decrease with surgeon familiarity [35]. Furthermore, specific complications have been reported, including lateral femoral cutaneous nerve (LFCN) injury [20, 36–38], fractures of the greater trochanter or femoral shaft due to high soft-tissue tension during femoral preparation, and even ankle fractures associated with the use of traction Table [39–42].

Our modification of the DAA addresses several of these critical issues. By eliminating the use of a traction table and avoiding specialised instrumentation, this variant simplifies the procedure and removes the need for additional dedicated staff in the operating room. Importantly, by freeing the lower limbs from traction, this approach reduces the risk of traction-related complications such as ankle fractures and decreases the risk of iatrogenic femoral fractures by lowering the tension exerted on periarticular soft tissues. In our experience, the absence of traction table manoeuvres and dedicated instrumentation also translates into shorter operative times, overcoming one of the most frequently cited drawbacks of the DAA in the literature. Moreover, the technique has proven versatile and reproducible across all patient phenotypes, including obese and muscular individuals, and can be extended with appropriate modifications to revision cases and complex deformities.

## Conclusion

This simplified variant of the DAA preserves the benefits of the anterior approach, such as reduced muscle damage and faster early recovery, while addressing many of its limitations. By reducing operative time, avoiding dedicated equipment, and minimising the risk of approach-specific complications, this technique represents a safe, effective, and accessible option for primary and revision total hip arthroplasty. Surgeons adopting this variant should still undergo appropriate training and be familiar with the local anatomy, but its streamlined workflow facilitates broader applicability and safer implementation in diverse clinical settings.

## Supplementary Information

Below is the link to the electronic supplementary material.

Supplementary Material 1

## Data Availability

No datasets were generated or analysed during the current study.
