# Supplementary material for: A modified direct anterior approach for primary total hip arthroplasty: surgical technique
Source: J Orthop Surg Res. 2025 Oct 22;20:919. doi: 10.1186/s13018-025-06397-5 (PMC12542394; doi:10.1186/s13018-025-06397-5)
Supplement: Supplementary file 1 — Supplementary Material 1 [file 13018_2025_6397_MOESM1_ESM.docx]

# Compliance with SUPER Guidelines

| Item SUPER | Covered? | Explanation |
| --- | --- | --- |
| 1. Title/Abstract | Covered | The title clearly identifies the technique as a simplified variant of the direct anterior approach (DAA) for THA; the abstract summarizes the rationale, objectives, and main features. |
| 2. Background/Rationale | Covered | Background and rationale are fully explained: current limitations of standard DAA with traction tables and dedicated instruments, and the need for a simpler, reproducible technique. |
| 3. Objectives | Covered | Objectives are explicitly stated: to provide a highly reproducible and safe surgical technique, minimizing complications and broadening applicability. |
| 4. Classification of the technique | Covered | Already specified in the title and text: this is a simplified variant of the direct anterior approach for primary THA, extendable to revisions. |
| 5. Name of the technique | Covered | Clearly indicated throughout the manuscript as 'modified direct anterior approach (DAA)'. |
| 6. Setting/Environment | Covered | Well described: no traction table required, no dedicated staff, three surgeons as operating team. Standard operating room suffices. |
| 7. Required equipment/instruments | Covered | No dedicated or exclusive instrumentation required. All instruments are widely available in orthopedic theaters. |
| 8. Personnel/Team | Covered | Standard surgical team; no need for additional staff for traction table management. |
| 9. Patient selection/indications | Covered | The technique is feasible in all patient phenotypes, including obese and muscular patients, and can be applied to revisions and complex cases. |
| 10. Contraindications | Covered | No additional contraindications beyond those of standard THA; obese and muscular patients are not excluded. |
| 11. Patient preparation | Covered | Preoperative positioning and skin preparation are detailed. |
| 12. Key landmarks and anatomy | Covered | Anatomical landmarks are carefully described step-by-step and reinforced with figures. |
| 13. Step-by-step description | Covered | Detailed, structured description of each surgical phase is provided (incision, exposure, osteotomy, acetabular and femoral preparation, reduction, stability tests). |
| 14. Variations/alternatives intraoperatively | Covered | Variants and technical alternatives are reported within the step-by-step description and surgical tips. |
| 15. Safety issues/precautions | Covered | Safety considerations are highlighted within the procedural description and surgical tips (nerve preservation, prevention of fractures). |
| 16. Quality control (intraoperative) | Covered | Intraoperative stability tests are performed in all planes; postoperative radiographic verification is systematically conducted. |
| 17. Postoperative tasks/criteria of success | Covered | Success criteria include immediate mobilization without restrictions, absence of dislocation, and early return to ambulation. |
| 18. Postoperative monitoring | Covered | Postoperative management is clearly described: immediate weight-bearing, no movement restrictions, standard rehabilitation pathway, scheduled follow-up visits. |
| 19. Complication management | Covered | Only LFCN neuropraxia observed, rare in our series, always resolving spontaneously with conservative treatment. |
| 20. Strengths/limitations | Covered | Strengths: reduced approach-specific complications, higher reproducibility, wider applicability. Limitation: learning curve, as with standard DAA. |
| 21. Perspectives/Future directions | Covered | The technique is extendable to complex deformities and revisions, supporting future broader application and multicentre validation. |
| 22. Other information | Covered | Ethical compliance, contributions, conflicts of interest, and explicit statement of adherence to SUPER guidelines are included. |
